# Diet supplementation with hemp (*Cannabis sativa L.*) inflorescences: effects on quanti-qualitative milk yield and fatty acid profile on grazing dairy goats

**DOI:** 10.1080/01652176.2024.2388715

**Published:** 2024-08-09

**Authors:** Ruggero Amato, Marianna Oteri, Biagina Chiofalo, Fabio Zicarelli, Nadia Musco, Fiorella Sarubbi, Severina Pacifico, Marialuisa Formato, Pietro Lombardi, Federica Di Bennardo, Piera Iommelli, Federico Infascelli, Raffaella Tudisco

**Affiliations:** a Department of Veterinary Medicine and Animal Production, University of Napoli Federico II, Naples, Italy; b Department of Veterinary Sciences, University of Messina, Messina, Italy; c Institute for the Animal Production System in the Mediterranean Environment, National Research Council, Portici, Italy; d Department of Environmental, Biological and Pharmaceutical Sciences and Technologies, University of Campania Luigi Vanvitelli, Caserta, Italy

**Keywords:** Hemp, dairy goats, fatty acid profile

## Abstract

Hemp (*Cannabis sativa L*.) is an annual plant belonging to the family of Cannabaceae with several varieties characterized by different fatty acid profile, content in flavonoids, polyphenols, and cannabinoid compounds. Hemp is mostly used in livestock nutrition as oil or as protein cake, but not as inflorescences. The aim of this study was to evaluate the effect of dietary hemp inflorescences on milk yield and composition in grazing dairy goats. Twenty Camosciata delle Alpi goats at their 3rd parity and with a mean body weight of 45.2 ± 2.0 kg, immediately after kidding, were equally allocated into two groups (G: Grazing and GH: grazing and hemp). For three months, all goats were fed on a permanent pasture and received 700/head/day of concentrate; diet of group GH was supplemented with 20 g/head/day of hemp inflorescences. Goats’ body weight did not change during the trial. Individual milk yield was daily recorded and samples collected every 20 days for chemical composition and fatty acid profile analysis. No significant differences were found for milk yield and chemical composition. Caproic (C6:0) (1.80 *vs.* 1.74%; *p* < 0.01) and lauric acids (C12:0) were significantly higher in milk of group GH (4.83 *vs.* 4.32%; *p* < 0.01) as well as linoleic (C18:2) (2.04 *vs.* 1.93%; *p* < 0.05), adrenic acid (C22:4) (0.046 *vs.* 0.031%, *p* < 0.05), omega-6/omega-3 ratio (3.17 *vs.* 2.93, *p* < 0.05) and total conjugated linoleic acids (CLAs) (0.435 *vs.* 0.417%; *p* < 0.01). The results of this study suggest that the supplementation of grazing goats’ diet with hemp inflorescences may enhance the milk nutritional characteristics by increasing its content of CLAs and other beneficial fatty acids.

## 1. Introduction

The use of plants, herbs, vegetables, and their by/co-products, as alternative feeding strategies are gaining increased interest (Infascelli et al. 2021; Musco et al. 2022; Tufarelli et al. 2023).

Hemp (*Cannabis sativa L.*) is an annual plant of the family Cannabaceae which grows roughly anywhere and it includes various botanical subspecies (Żuk-Gołaszewska et al. 2018). *Cannabis sativa* var. *sativa* has gained particular interest in the last years for its low content in tetrahydrocannabinol (THC) (<0.2–0.3%), a chemical compound responsible for some psychoactive effects, which makes this cultivar suitable for legal cultivation in Europe (EFSA 2011). In fact, the European Food Safety Authority (EFSA) has allowed, since 2011, hempseed and hempseed cake (HSC) as feed ingredients for all the animal species.

For its nutritional characteristics this plant has been studied as feasible feed supplement in various animal species (Klir et al. 2019; Vastolo, Calabrò, et al. 2021; Vastolo, Iliano, et al. 2021). In particular, hemp is characterized by a great content in polyunsaturated fatty acids (PUFA) reaching almost the 80% of total fatty acids (FA), manly linoleic (LA), and alpha-linolenic acids (ALA) (Parker et al. 2003; Da Porto et al. 2012; Rapetti et al. 2021). Feeding animals with sources of PUFA enhances the concentration of beneficial FA in animal products with positive effects on human health (Kapoor et al. 2021). In this regard, LA and ALA represent the main substrate for rumen biohydrogenation (Griinari et al. 1999) which leads to the production of intermediated by-products in milk, such as conjugated linoleic acids (CLAs) known for their anticarcinogenic properties (Pipoyan et al. 2021). Additionally, hemp is also rich in bioactive compounds, such as natural antioxidants (polyphenols, tocopherols, carotenoids, and phytosterols) (Farinon et al. 2020), which according to Poulopoulou et al. (2020), increase the milk concentration of fatty acids beneficial for the consumers.

The most used parts of the hemp in livestock nutrition are the seeds (Rapetti et al. 2021) and the protein cake, a by-products obtained by squeezing the seeds for oil extraction (Serrapica et al. 2019) that has been demonstrated to be an alternative protein source. The whole hemp plant after cannabinoid extraction (spent hemp) has also been tested in dairy cows (Irawan et al. 2023) and in lambs (Parker et al. 2022) showing improved productive performances in supplemented animals. In a recent review (Altman et al. 2023) the use of industrial hemp as supplementation in livestock nutrition has been deeply explored. The authors suggest hemp seeds and inflorescences being the most promising animal health additive for their high content in cannabinoids that represent, at the same time, also the major concern for their safety in the food industry.

In fact, according to EFSA (2011), feed derived from the whole hemp plant could be used in animal diets when they do not contain more than the maximum legal THC concentration, even if dried hemp leaves and flowers should not be used in animal feeding.

To contribute to increase the knowledge on hemp derived products in animal nutrition, in this work hemp inflorescences have been used in grazing dairy goats and the milk obtained from the supplemented group was only destined to research.

The objectives of this work were (i) to test the effects of supplementing diet of goats with hemp inflorescences on milk composition and (ii) to evaluate the milk nutritional quality by studying the FA profile.

The hypothesis was that the beneficial compounds present in *Cannabis sativa* could improve milk nutritional characteristics.

## 2. Materials and methods

### 2.1. Animals and diet

The trial was performed according to the Animal Welfare and Good Clinical Practice (Directive 2010/63/EU) and was approved by the local Animal Ethics Committee (protocol number: PG/2019/0070006) at the ‘Funky Farm’, located in Sant’Apollinare (FR, Italy) at 400 m a.s.l. (41° 14′ N; 13° 50′ E), with an average rainfall of 530 mm and an average annual temperature of 6–23 °C.

Twenty multiparous goats [(Camosciata delle Alpi breed; body weight (BW) 45 kg (±2.0 kg)], were equally assigned into two groups (G: grazing; GH: grazing and hemp) homogeneous for parity (3rd) and days in milk (DIM: 60 ± 7). The trial started 60 days after kidding, as the milk produced during this period is typically reserved for the kids as local tradition. Goats’ energy requirements were calculated as follows: for maintenance 0.0365 UFL/kg metabolic weight (MW:BW^0.75^); for milk production 0.41 UFL/kg fat-corrected milk (FCM, 4% fat). Therefore, total energy requirement was 1.48 UFL (0.63 UFL maintenance + 0.85 UFL milk production) and since the average pasture DM intake in the inlands of South Italy is 20 g/kg BW (Iommelli et al. 2022) the deficit of 0.78 UFL was met by the concentrate. Body weight was measured fortnightly for each goat.

Both groups had free access to water and to a permanent pasture (8:00 am to 3:00 pm and 5:00 pm to 20:00 pm), mainly composed by the spontaneous vegetation of the area (*Trifolium alexandrinum*, *Vicia* spp., *Crataegus monogyna*, *Rubus ulmifolius*, *Clematis vitalba*, *Medicago sativa*, *Festuca arundinacea*, *Bromus catharticus*, *Festuca arundinacea*, and *Lolium perenne*). Goats were hand milked twice a day (7:00 am and 20:00 pm) and at the evening milking were placed in individual pen only for the feeding time. They received 700 g/head/day of concentrate composed by barley (23%), oats (22%), and faba beans (55%); group GH received in addition 20 g/head/day of Cannabis sativa Kompolti inflorescences.

The hemp inflorescences used in this trial were produced by the ‘Centro Sviluppo Canapa del Sud’ in Campania, Italy. This variety originates from Hungary, and in Italy is mainly cultivated for cannabinoid extraction (Ovidi et al. 2022). Inflorescences were gathered at a flowering stage and then dried at 21–23 °C for 10 days; subsequently, the flowers were trimmed.

### 2.2. Feed sampling and analysis

To reduce the differences in chemical composition observed in studies based on a monthly collection (D’Urso et al. 2008; Tudisco et al. 2019b), in this trial the pasture samples were weekly collected. Every week (from May to July) pasture was sampled from four different areas measuring 2.5 m^2^, cutting at 3 cm from the ground, and then, after oven dried at 65 °C and weighing, the 4 weekly representative samples (1 kg each obtained balancing the amount from the 4 different areas) were pooled for a monthly analysis. Hemp inflorescences and concentrate were sampled in triplicate only at the beginning of the trial. Pasture samples were milled through a 1 mm screen, as well as concentrate and hemp samples, and analyzed for chemical composition according to AOAC (2012) whereas the structural carbohydrates were determined as suggested by Van Soest et al. (1991) and nutritive value (UFL = 1700 kcal of net energy for lactation) was calculated according to INRA et al. (2018). Feed fatty acid (FA) profile was determined by gas chromatography (GC).

Around 200 mg of fat, extracted according to Folch et al. (1957), were directly methylated as suggested by Christie (1993). In brief, the lipid extract was added with 2 mL of a methanol:sulfuric acid (9:1, v/v) mixture, heated in air-oven (100 °C for 1 h), cooled at room temperature, and added with 1 mL of n-hexane. Successively, the solution was filtered (0.45 μm filter) and injected into a GC with flame ionization detector (FID). The analyses of fatty acid methyl ester (FAME) were performed by a GC-FID (TRACE 1310) system equipped with an AI 1310 Auto-injector/AS 1310 Autosampler (Thermo Fisher Scientific, Milan, Italy). A capillary polar column Omegawax 250 (Supelco, Bellefonte, PA, USA), 30 m × 0.25 mm, 0.25 μm (L × I.D., film thickness) was adopted with the following oven temperature program: 100 °C for 5 min, 100–240 °C at 4 °C/min, final isotherm at 240 °C for 20 min. Injector and detector temperature, 250 °C; injection volume, 0.5 µL; split ratio, 1:50. Carrier gas (He) at a flow rate of 1 mL/min; make-up gas (N2) flow, 40 mL/min; H2 flow, 35 mL/min; air flow, 350 mL/min. Data were processed with the Chromeleon™ Data System (Thermo Fisher Scientific, Milan, Italy) software (Version 7.2.9) and individual FAMEs identified by comparing sample peak retention times with standards from Supelco (Merck KGaA, Darmstadt, Germany). Single FA concentrations were expressed as g/100 g, considering 100 g the total of all areas of the identified FAMEs.

### 2.3. Milk sampling and analysis

From May to July 2021, individual milk yield was daily recorded and individual milk samples, obtained by weighting the morning and the evening milkings, were collected every 20 days (a total of five sampling). Milk chemical composition was evaluated using Milko Scan 133B (Foss Matic, Hillerod, Denmark) standardized for goat milk.

Milk FA profile was determined by extraction of total fat with a hexanopropanol and isopropanol (3/2 v/v) (Hara and Radin 1978) mixture and subsequent trans methylation (Christie 1982) modified by Chouinard et al. (1999).

The methyl esters were quantified by GC (ThermoQuest 8000TOP gas chromatograph, Thermo Electron Corporation, Rodano, Milan) with flame ionization detector and with capillary column (CP-SIL 88 fused silica capillary column, 100 m × 0.25 mm internal diameter with 0.2-μm film thickness; Varian, Inc., Walnut Creek, CA, USA) adopting the following temperature ramp:

$$\begin{array}{l} 70{}^{\circ}\text{ C for 4 min}\to 13{}^{\circ}\text{ C}/\text{min}\to 175{}^{\circ}\text{ C for 27 min}\\ \;\to 3{}^{\circ}\text{ C}/\text{min}\to 215{}^{\circ}\text{ C for 38 min}\to 10{}^{\circ}\text{ C}/\text{min}\to 70{}^{\circ}\text{ C}. \end{array}$$

The temperature of injector and detector were at 250 and 260 °C, respectively. Gas flows were: carrier gas (helium) 1 ml/min; hydrogen 30 ml/min; air 350 ml/min; make-up gas (helium) 45 ml/min.

FA peaks were identified by comparing with a standard mixture of fatty acid methyl esters (Larodan Fine Chemicals, AB, Limhamnsgårdens Malmö, Sweden). CLA isomers were identified by comparing samples chromatograms with those of single purified isomers (CLA cis-9, trans-11; CLA trans-10, cis-12; CLA cis-9, trans-11; CLA trans −9, trans-11) (Larodan Fine Chemicals, AB, Limhamnsgårdens Malmö, Sweden).

### 2.4. Statistical analysis

Feed data were analyzed using the one-way ANOVA with JMP software (version 11, PROC GLM, SAS 2000) according to the following model:

Yij=μ+Si+εj

where Yij = mean of response variable, μ = general mean, Si = sampling effect (*i* = 3; May, June, July), and εj = experimental error.

Body weight and milk data were analysed using the two-way ANOVA for repeated measures with JMP software (version 11, PROC GLM, SAS 2000) according to the following model:

Yijk=μ+Di+Sj+(DS)ij+εijk

where: y = mean of response variable, *μ* = general mean, Di = effect of the dietary treatment (*i* = 2; G and GH), Sj = sampling effect (j milk = 5; I, II, III, IV, V), DS = interaction between dietary treatment × sampling effect, and εijk = residual error. The means were statistically compared using Tukey’s test. Differences were considered statistically significant at *p* < 0.05.

## 3. Results

No refusals were detected. Feeds chemical composition is reported in Table 1. Hemp was characterized by a high content of crude protein and ash.

**Table 1.** Chemical composition (g/kg DM; mean ± *SD*) and energy value of feed.

	Pasture	Concentrate	Hemp
Crude protein	159.0 ± 1.2	186.0 ± 1.7	261.3 ± 1.0
Ether extract	23.0 ± 0.3	34.2 ± 1.8	28.0 ± 0.2
NDF	487.1 ± 5.6	267.1 ± 3.3	405.9 ± 3.2
ADF	351.0 ± 2.7	108.1 ± 1.1	310.0 ± 1.9
ADL	53.2 ± 1.7	36.3 ± 0.9	163.3 ± 2.4
Ash	69.8 ± 1.4	11.2 ± 1.3	162.8 ± 2.4
UFL/kg DM	0.78	1.1	0.78

NDF: neutral detergent fiber; ADF: acid detergent fiber; ADL: acid detergent lignin; UFL: unit feed for lactation.

Feeds FA profiles are reported in Table 2. Both pasture and hemp had a high value in PUFA, especially in omega-6 and also omega-3. Pasture and hemp had an opposite ratio between n6/n3, anyway guaranteeing both a significant amount of substrates for rumen biohydrogenation.

**Table 2.** Fatty acid profile of hemp and pasture during the trial (% of total FA).

	Concentrate	Hemp	Pasture		
			May	June	July
SFA	22.9 ± 0.8	10.1 ± 0.2	18.4 ± 0.2	17.2 ± 0.8	19.6 ± 0.5
MUFA	25.4 ± 1.1	17.0 ± 0.5	4.0 ± 0.1	4.0 ± 0.1	4.1 ± 0.1
PUFA	51.7 ± 3.3	70.1 ± 1.3	76.8 ± 1.0^B^	79.4 ± 0.9^A^	75.7 ± 0.8^B^
LA	45.8 ± 2.8	43.9 ± 0.1	23.1 ± 0.17^B^	33.7 ± 0.5^A^	20.4 ± 0.2^B^
ALA	2.8 ± 0.2	26.3 ± 0.1	41.4 ± 0.1^B^	42.8 ± 0.2^A^	41.1 ± 0.2^B^
Omega-6	46.6 ± 3.0	44.2 ± 0.5	23.7 ± 0.21^B^	34.2 ± 0.5^A^	20.9 ± 0.4^B^
Omega-3	3.1 ± 0.2	26.7 ± 0.1	43.0 ± 0.2^B^	44.2 ± 0.3^A^	43.0 ± 0.2^B^
Omega-6/omega-3	15.0	1.6	0.5^B^	0.8^A^	0.5^B^

SFA: saturated fatty acids; MUFA: monounsaturated fatty acids; PUFA: polyunsaturated fatty acids; LA: linoleic acid; ALA: alpha-linolenic acid.

A and B in superscript within the rows differ significantly (*p* < 0.001).

Goats BW did not significantly change during the trial. Milk yield was not significantly different between the groups (g/head/day 2336 *vs.* 2276, for group GH and G, respectively). Similarly, no differences were found in milk composition (Table 3).

**Table 3.** Goats’ body weight (kg), milk yield (g/d), and chemical composition (%).

	Treated group	Control group	RMSE
Body weight (kg)	45.3	45.2	5.10
Milk yield	2336	2276	663.8
Protein	3.39	3.39	0.179
Fat	3.68	3.63	0.438
Lactose	4.91	4.86	0.252

Treated group: group GH; Control group: group G; RMSE, root mean square error.

In Table 4, the mean values of milk FA are reported. Among saturated fatty acids (SFA), caproic (C6:0) and lauric acid (C12:0) were significantly higher (*p* < 0.001) in group fed with hemp, whereas among the unsaturated fatty acids, trans vaccenic acid (C18:1 trans 11) and alpha linolenic (C18:3) acid were significantly lower (*p* < 0.001) in group GH, while adrenic acid (AdA; C22:4) was significantly higher (*p* < 0.001) compared to group G.

**Table 4.** Milk fatty acids (% of total FA).

	Treated	Control	Group effect	Time effect	Interaction	RMSE
C4:0	1.434	1.566	0.038	<0.0001	0.01	0.308
C6:0	1.809	1.742	<0.0001	<0.0001	<0.0001	0.245
C8:0	2.967	2.935	0.598	<0.0001	<0.0001	0.303
C10:0	11.77	11.54	0.262	<0.0001	<0.0001	0.968
C11:0	0.195	0.177	0.08	0.416	0.0028	0.049
C12:0	4.828	4.391	0.0003	0.0134	0.0007	0.575
C13:0	0.056	0.047	0.0365	0.0223	0.0013	0.021
C14:0	10.33	10.14	0.077	0.003	0.287	0.517
C14:1	0.077	0.079	741	<0.0001	0.258	0.031
C15:0	0.451	0.447	0.846	0.348	0.136	0.093
C16:0	26.85	27.74	0.0149	<0.0001	0.958	1.754
C16:1	0.223	0.247	0.033	<0.0001	0.014	0.054
C17:0	0.339	0.372	0.019	0.01	0.053	0.066
C17:1	0.029	0.027	0.407	<0.0001	0.64	0.014
C18:1 CIS6	0.043	0.030	0.04	0.069	0.129	0.03
C18:0	14.82	14.88	0.938	0.0008	0.042	1.465
C18:1 trans 9	0.258	0.299	0.012	<0.0001	0.778	0.078
C18:1 trans 11 (TVA)	1.69	2.06	<0.0001	0.0041	0.025	0.414
C18:1 CIS9	16.47	16.11	0.257	0.0082	<0.0001	1.538
C18:1 CIS10	0.334	0.348	0.501	<0.0001	0.027	0.093
C18:1 CIS11	0.308	0.359	0.0016	<0.0001	0.207	0.076
C18:1 CIS12	0.125	0.138	0.078	<0.0001	0.014	0.034
C18:2 trans N6	0.126	0.138	0.212	<0.0001	0.044	0.046
C18:2 CIS N6 (LA)	2.04	1.93	0.047	<0.0001	0.262	0.267
C20:0	0.167	0.165	0.736	0.0243	0.026	0.024
C18:3 N6	0.032	0.040	<0.0001	0.0116	0.0006	0.009
C20:1	0.036	0.036	0.826	0.0003	0.011	0.01
C18:3 N3 (ALA)	0.754	0.792	0.166	<0.0001	0.738	0.133
C18:2 cis 9 trans 11	0.286	0.279	0.671	<0.0001	0.047	0.085
C18:2 trans 10 cis 12	0.148	0.141	0.28	<0.0001	0.005	0.033
C20:2 N6	0.028	0.027	0.733	0.0054	0.0028	0.0084
C22:0	0.067	0.064	0.405	<0.0001	0.101	0.0189
C22:1	0.013	0.017	0.014	<0.0001	0.023	0.0076
C20:3 N3	0.011	0.01	0.664	<0.0001	0.386	0.0058
C20:4 N6 (AA)	0.096	0.105	0.075	<0.0001	<0.0001	0.024
C22:2 N6	0.017	0.015	0.448	0.023	0.308	0.0123
C24:0	0.016	0.016	0.709	<0.0001	0.844	0.0039
C20:5 N3 (EPA)	0.048	0.05	0.63	<0.0001	0.002	0.0135
C24:1	0.02	0.021	0.829	0.249	0.041	0.0083
C22:4 N6	0.046	0.031	<0.0001	0.0001	0.763	0.0143

Treated group: group GH; Control group: group G; RMSE. root mean square.

The FA classes and the nutritional indices are reported in Table 5. The total CLA content was higher in group fed with hemp (*p* < 0.001), as well as the omega-6/omega-3 and the LA/ALA ratio (*p* < 0.05 and *p* < 0.01, respectively).

**Table 5.** Milk fatty acid classes and ratios.

	Treated	Control	Group effect	Time effect	Interaction	RMSE
SFA	76.127	76.278	0.6996	0.068	0.0004	1.9078
MUFA	19.627	19.722	0.786	0.0221	<0.0001	1.718
PUFA	3.643	3.575	0.436	0.288	0.97	0.42
Omega-6	2.395	2.304	0.122	<0.0001	0.471	0.286
Omega-3	0.812	0.854	0.121	<0.0001	0.673	0.132
ΣCLA	0.435	0.417	<0.0001	<0.0001	0.026	0.11
PUFA/SFA	0.047	0.047	0.523	0.406	0.777	0.0064
Omega-6/omega-3	3.17	2.93	0.016	<0.0001	0.113	0.493
LA/ALA	2.96	2.68	0.005	<0.0001	0.091	0.477
AA/EPA	2.15	2.32	0.318	0.007	0.805	0.839

Treated group: group GH; Control group: group G; RMSE: root mean square; SFA: saturated fatty acids; MUFA: monounsaturated fatty acid; PUFA: polyunsaturated fatty acids; CLA: conjugated linoleic acid; LA: linoneic acid; ALA: alpha-linolenic acid; AA: arachidonic acid; EPA: eicosapentanoic acid.

## 4. Discussion

Body weight didn’t change during the trial in both the experimental groups. The diet of all the grazing goats were formulated to satisfy their energy requirements according to Tudisco et al. (2014).

Pasture nutritional characteristics are similar to those reported in previous studies on spontaneous pasture of Mediterranean areas (Cabiddu et al. 2005; Tudisco et al. 2012; Lo Presti et al. 2023). The most abundant fatty acid was C18:3 and, in contrast to Meľuchová et al. (2008), it remains high during the trial. Indeed, Meľuchová et al. (2008) reported a decrease in ALA content from May to July and an increase in LA that we only detected in June. As those authors suggested, the differences in ALA and LA content are mainly due to the different plants composing the pasture or by their phenological phase (Cabiddu et al. 2005).

With regards to hemp inflorescences’ nutritional characteristics, the content in crude protein (CP: 26.1%), comparable to that of leguminous hay, makes this plant suitable for animal nutrition (Bailoni et al. 2021) as for its high content in PUFA (70%). Hemp inflorescences had high content of ash, as also reported by other studies (Vastolo, Calabrò, et al. 2021; Altman et al. 2023; Irawan et al. 2023); this could be due to the harvesting and processing techniques (McDonald et al. 2011).

Inflorescences fatty acid profile is characterized by a high content in linoleic acid (LA: 43.9%) and ALA (26.3%). Similar results of LA were reported by Piovesana et al. (2021) in hemp inflorescences and by Karlsson et al. (2010), Mierlita (2018), and Rapetti et al. (2021) in the hemp cake and seeds. These fatty acids are of particular interest in animal nutrition because they are substrates of rumen biohydrogenation for CLAs synthesis (Chilliard et al. 2007).

In the present study, the milk yield was not increased by hemp supplementation according to Irawan et al. (2023) which administered the whole spent hemp plant to dairy cows.

Moreover, no changes in milk composition were registered, in contrast to the results obtained by other authors which used hemp seeds (Karlsson et al. 2010; Cozma et al. 2015; Cremonesi et al. 2018; Mierlita 2018), and observed significantly changes in milk protein and fat content in the hemp supplemented group. In particular, Karlsson et al. (2010) evaluated in dairy cows four diets with increasing levels of hempseed cake and observed significant positive effects on the milk yield, while milk protein and fat decreased, with a 14% inclusion of hempseed cake (DM basis) in the diet. Mierlita (2018) obtained increased milk yield and milk fat content by using both hempseeds or hemp cake in the diet of sheep while Cozma et al. (2015) and Cremonesi et al. (2018) reported significant increase in milk fat in goats receiving a diet supplemented with hempseed or hempseed oil, respectively.

Nevertheless, nutritional characteristics of milk obtained by both groups were favorable to human health for their low omega-6/omega-3 ratio (Simopoulos 2022). This result is probably due to the use of pasture in both groups, which is rich in omega-3 and provides a great quantity of other beneficial fatty acids in ruminants diet and in their derived products (Cavaliere et al. 2018; Trinchese et al. 2019; Mollica et al. 2021).

Lauric acid (C:12) was significantly increased in milk of GH group in contrast to Rapetti et al. (2021). Lauric acid is a medium chain SFA which is mainly derived by endogenous synthesis, which can be inhibited by the abundance of long chain fatty acids in the diet (Chilliard et al. 2000). In the recent years, several authors have shown the numerous potentialities of lauric acid in *in vitro* studies (DiNicolantonio and O’Keefe 2017; Tham et al. 2020; Verma et al. 2020), demonstrating its antidiabetic and anti-insulin resistance effects and its efficacy in inhibiting cancer cells proliferation.

With regards to the milk fatty acid classes, other authors reported a significant increase in trans-vaccenic, alfa-linolenic, eicosapentaenoic, docosahexaenoic acids and in total CLA content (Mierlita 2016) whereas a reduction in neo synthesis fatty acids was observed (Cremonesi et al. 2018; Rapetti et al. 2021) when different source of hemp are used as supplement. Cozma et al. (2015) reported a significant increase of milk PUFA in goats supplemented by hempseed oil, without an effect on omega-3 fatty acids content.

Among PUFA, we observed significantly higher content of linoleic acid (*p* < 0.05) and total CLA (*p* < 0.0001) in treated group. These increases could be explained by the great content of omega-6 in hemp inflorescences which directly enriches the milk and at the same time acts as substrate for rumen biohydrogenation increasing the total content of milk CLA isomers widely known for their anticancer properties as well as for contrasting inflammation and reducing the risk of cardiovascular diseases (Griinari et al. 1999).

Of particular interest is the content of adrenic acid (C22:4), a long chain PUFA, which had significantly higher levels (*p* < 0.001) in GH group. The role of adrenic acid on human health is controversial; recent studies demonstrated its protective effect against the risk of depression (Zeng et al. 2022) and its anti-inflammatory effects (Brouwers et al. 2020) while Zhao et al. (2022) demonstrated its oxidative role in hepatocytes.

## 5. Limitation of the study

The two different diets administered to the goats were not formulated for being isonitrogenous and isocaloric, representing a limitation of this study. Further studies are therefore needed to evaluate different level inclusion of hemp inflorescences in isonitrogenous diet to obtain beneficial effects.

## 6. Conclusions

The aim of this study was to evaluate the effect of hemp inflorescences supplementation in the diet of grazing goats on milk yield, composition, and fatty acid profile. Our results revealed that hemp supplementation affects milk fatty acid composition, increasing the total content of CLAs and of other beneficial FA, therefore suggesting that this plant could be potentially used in animal diet to improve milk quality. Further studies are needed to evaluate different levels of inflorescences supplementation and to investigate the aromatic characteristics of the product obtained. Moreover, the great attention that hemp had gained in the last years offers the possibility to use the different products that the cultivation of this plant offers in animal production.
